# Investigation on an Absorbing Layer Suitable for a Noise-Reducing Two-Layer Pavement

**DOI:** 10.3390/ma13051235

**Published:** 2020-03-09

**Authors:** Sabine Faßbender, Markus Oeser

**Affiliations:** Institute of Highway Engineering, RWTH Aachen University, Mies-van-der-Rohe-Str. 1, 52074 Aachen, Germany; oeser@isac.rwth-aachen.de

**Keywords:** low-noise, polyurethane, porous pavement, crumb rubber, asphalt performance, polyurethane-bound materials, absorption, sustainable pavement

## Abstract

A polyurethane-based rubber-modified layer within a road superstructure leads to absorption of traffic emissions. Noise emissions have quite a negative effect on society, as they lead to high stress levels and health risks for people. Therefore, constructional methods of noise-reducing road layers have been developed before. This research paper focuses on the questions whether the existing noise-reducing road constructions, which have a low durability, can be optimized in terms of a longer duration while simultaneously maintaining the noise-reducing effects. Within this research, a large parametric study contributed to an optimal solution of a noise-reducing and durable layer. We found that noise absorption is mainly dependent of the void content of the pavement and its flexibility. Also, a result is that the durability of a road layer is based on the properties of the binder as well as the composition of the mixture, i.e., the grading curve. As we used polyurethane binders within our mixtures, which have a low dependency on regular environmental temperatures after their complete chemical reaction, we can imply a low temperature dependence of the entire polyurethane asphalt mixture. Based on these results, the construction of a noise-reducing and durable road layer is a great solution. The application of such road layers leads to lower traffic emissions at major hotspots. These might be urban highways, where the infrastructure is too tight to build noise barriers, enclosures or tunnels.

## 1. Introduction

Traffic noise is a negative side effect that is constantly growing due to the increasing volume of traffic. For this reason, the reduction of traffic noise is the subject of long-term research, which must always be further developed. Within the scope of this topic, [1] developed a two-layer road surface system for noise reduction. The aim of the surface course system was to reduce the noise caused by road traffic by developing new artificial materials. This was achieved by combining a two-layer surface course system. The surface course system consists of a drivable thin layer of polymethyl methacrylate and fillers, and has a special texturing that greatly reduces the development of tyre/road noise (wearing course). The wearing course consists of a surface that represents a concave shape. A concave surface occurs in all roller compacted surfaces and forms an even road surface with gaps. This shape of the surface reduces the excitation of the tyres to vibrate and thus reduces the development of tyre–road noise. Air compressions are also avoided, as the formed gaps allow great air drainage. Further, it is designed to be permeable, so that any noise that continues to be generated can pass through it to the bottom layer. The bottom layer is a polyurethane-bonded layer of rubber particles and aggregates designed to absorb further noise [1,2]. In Germany, low-noise asphalt compositions which are bitumen-bound and do not have sufficient durability are common. This led to the idea of using new materials such as polyurethane, which were known to be suitable for specific material behavior by adjusting their chemical composition. While [1] mainly focused on the absorption properties of the system, this study focuses on the development of durability while maintaining the noise-reducing effect [2].

### 1.1. Performance of Noise Absorption through Road Layers

For the acoustics of road top layers, the type of surface course, the construction method, the mix composition and the surface design are of importance. As soon as a road surface layer is open-graded, i.e., a porous asphalt or a drain concrete, the porosity of the surface allows traffic noise to pass the complex cavity system of the layer. This cavity system leads to an absorption of incoming sound waves. The structure and the cavity size of the cavity system decisively influence the sound absorption capacity of the layer [3].

The particle size distribution and the selection of the binder type as well, as the thickness of the noise absorbing asphalt layer, are designed in a way that the cavity system is as pronounced as possible. In principle, the fine fractions of a mix formulation are not used for this purpose. However, the aim is to create a load-bearing construct. A single-stage grain mixture, such as the pervious pavements (PA) according to the German guideline TL Asphalt 07/13, enables such a load transfer due to the tilted aggregates underneath [3].

With a high porosity, it is possible to obtain approximately 100% absorption within a small frequency range [3,4]. Ref. [4] describes that for a conventional porous asphalt, in a narrow frequency range at 800 Hz almost 100% of the incident sound energy is absorbed. The absorption capacity depends on the position of the frequency range of the incoming sound waves. Depending on the thickness of the layer, different frequency ranges and frequency bandwidths can be absorbed [4].

In order to achieve a noise absorption of the roadway, it is necessary to obtain an exact knowledge of the type of vehicles driving over it. The traffic collective, consisting of cars and trucks, generates sound waves of different frequency ranges. These are due to different tyres and driving speeds. In order to maximise the absorption capacity of an asphalt layer, the maximum of the absorption frequency response of the layer must be superimposed with the spectral maximum of the tyre–road noise from the traffic collective [3].

From Beckenbauer [4] it is known that the highest frequency of the sound level from a traffic collective with low truck share and high speeds is about 1000 Hz. However, other traffic flows with a higher truck share and lower travel speeds generate a wider frequency spectrum with two peaks within the absorbing curve in the range of 500 Hz to 1000 Hz. Ref. [4] also refers to the sound power levels of tyre road noise and drive noise separately for passenger cars, light trucks and heavy trucks as a function of frequency and speed in his study. This shows that tyre–road noise (TRN) is mainly between 500 and 2000 Hz and has a maximum in the range 800 to 1250 Hz. With regard to the acoustic effectiveness of the absorption of a road surface, the structural parameters of the layer must be adapted to the existing traffic collective.

According to [4], the layer thickness of the asphalt layer determines the position of the frequency maximum in the frequency range between 0 Hz and 8000 Hz. Furthermore, the layer thickness is responsible for the number of absorbable frequency maxima. The height of the porosity, i.e., the cavity system accessible from the outside, determines the height of the amplitude of the frequency maximum, i.e., the maximum absorption capacity.

In addition, the flow resistance in the widely branched cavity system affects the width of the amplitude of an absorbable frequency maximum. It represents a delay of the sound waves when flowing through the pores of a body [5]. Flow resistance is the resistance against air flow through a body. It is significantly influenced by the course of the pore channels and therefore increases with the thickness of the body [6]. The main characteristic of absorbing materials is the friction in the pore channels, which reduces air resistance by converting the kinetic energy of the sound waves into heat, thereby depriving it of power [6].

According to [3,7], the stiffness of an absorber layer leads to a noise optimization. This is also known as the mechanical impedance effect. Comparative tests with the same texture of sandpaper and different layers below (concrete layer and elastic layer), Ref. [3] could determine differences in noise generation at traffic crossings. Ref. [7] confirms this phenomenon and justifies it with the fact that sound waves of two materials whose impedance values are in the same order of magnitude can be better transferred to the other medium compared to materials whose impedances distinguish large. In our context, this could clearly mean that the mechanical vibrations emanating from a tyre tread block can be better transferred to a rubber-containing substrate or can be damped and absorbed by it. If, on the other hand, the excited tyre tread blocks hit a hard asphalt layer, the sound excitation may be stopped abruptly and, in extreme cases, no transfer to the ground occurs [7].

### 1.2. Performance of Mechanical Behavior of Open-Pore Rubber Modified Asphalt

Besides of acoustic effectiveness, the road user expects road safety, driving comfort and absolute availability with regard to the infrastructure while driving his vehicle. This expectation requires the building load bearers to guarantee functional requirements such as grip, evenness and noise reduction as well as the construction requirements for roads such as load-bearing capacity and durability. In order to ensure these requirements, a comprehensive knowledge of the material properties of the road surface to be used is required. Road pavements must therefore meet performance requirements and be tested in terms of fatigue, deformation and low temperature behavior.

Open-pore asphalt cannot fully meet the demands placed on it. As a result, open-pore asphalt bound with bitumen often fails prematurely. Recent studies by [8,9,10,11,12,13] prove that the addition of rubber particles and additives or even the complete substitution of bitumen by alternative binders such as epoxy resin has a positive effect on the durability of open-pore asphalt. Refs. [8,9,10,11,13] show in various applications that the durability of the modified pavements is increased and the noise-reducing properties can thus be guaranteed over a longer period of time.

In order to improve the high temperature resistance of asphalt with rubber granules, Ref. [11] investigated and compared the effects of additives on rubber compound asphalt and found that additives, such as Granular Polymer Durable additive (GPDa), have higher fatigue life and sensitivity to fatigue cracking as well as a good rutting performance.

Since in reality it is not possible to adjust the road construction in such a way that it fulfils all properties to the maximum degree, the different characteristics with regard to the use of certain asphalt layers are weighed against each other during the asphalt design and adjusted and optimized to the local requirements. This adjustment of the overall construction and the individual layer is based on material laws that describe the material behaviour.

## 2. Materials and Methods

Main materials of this study are aggregates, polyurethane binders and crumb rubber. The coarse aggregate consists of basalt and a common limestone is used as filler. In addition to the mentioned components, rubber particles from shredded scrap tires are included in the mix. All aggregates were dried to a constant mass before the mixing process. The rubber particles were visually checked for moisture. As they were delivered in dry condition, they were added directly to the mixture. Subsequently, all components were mixed with the binding agent. The aim is to achieve the highest possible void content in elastic specimens in order to obtain the basic conditions for absorbing asphalt layers explained in Section 1.1. Aggregates and rubber particles are bonded with the single component elastified polyurethane adhesive Elastan* 6568/103 from BASF Polyurethanes GmbH (Lemförde, Germany).

The grading curve on which this study was initially based was the grading curve of a study by [1]. An international short overview of his study is available in [2]. Ref. [1] developed a two-layer road surface system, which has a noise-reducing effect. The system consists of two layers, a wearing course and an absorption layer. The latter is based on previous studies concerning poro-elastic road surfaces (PERS) ([14,15,16,17]) and shows deficits in its stability. Therefore, the layer is optimized in terms of durability using this study, as the previous developments ([1,14,15,16,17]) only offer maximum noise absorption but a far from adequate suitability for use. The absorption layer developed by [1] ([2]) was therefore further developed. The further development of the absorption layer is the basis of this publication.

A major problem with the use of PERS layers is their high flexibility, which means that the layer will exhibit higher deformation when loaded. This elasticity leads to a good absorption capacity in terms of noise, as the transmission of the resulting structure-borne noise in the layer is reduced by the rubber particles. It may be possible to use such a system as a single layer, even if the high deflections result in increased rolling resistance. This is a significant proportion of tyre resistance, along with frictional and aerodynamic resistance, and influences the energy consumption of the vehicle and the driving behavior of the user. Due to the constant deformation of the material, fatigue cracks can occur within the rubber particles or the binder matrix.

**Figure 1:**
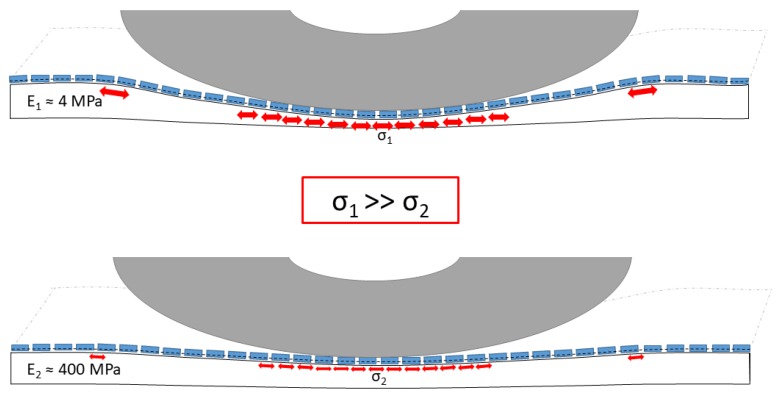
Exemplary representation of the stress input through the texture layer into the absorption layer at different stiffnesses of the substrate. (**Top**) high inclination of texture elements, (**bottom**) low inclination of the texture elements.

If the absorption layer is combined with a wearing course of very stiff material joined by a flexible reinforcement, the absorption layer must be protected from high stress through the edges of the texture layer elements. The stress input of an element that can be pressed into an elastic layer is highest when the absorption layer has maximum elasticity. If, on the other hand, the absorption layer is stiffer, the texture layer element cannot get into a high inclined position and presses itself less into the underlying layer, which leads to lower stress input (Figure 1).

### 2.1. Determination of the Decisive Elastic Modulus Considering the Impact of the Stiff Top Layer

Based on simulations that have been conducted in the project called INNO-PAVE [18], an elastic modulus of E_el_ = 300 MPa allows deflections of the absorption layer of 0.5 mm. Although this deflection is high compared to conventional asphalts, it significantly reduces the stress peaks in the underlying absorption layer. This allows the system to be still elastic, but not subject to the enormous stresses that are likely to cause the material to fail quickly.

Hence, the aim was to increase the stiffness of the absorption layer to such an extent that no critical sinking depths occur and at the same time the acoustic effectiveness is maintained. However, two target values are formulated:

1. The elastic modulus of the layer must meet the stiffness requirements of at least 300 MPa: $E_{el}\geq$ 300 MPa.
2. The sound absorption capacity in the frequency range of the tyre–road-noise (TRN) (800 to 1250 Hz) should be as high as possible: $\alpha_{TRN}\to max$.

### 2.2. Approach and Test Matrix

Noise reduction through technical road surface construction measures refers to changes in the texture, porosity and flexibility of the road surface. Since in this case it is not a directly trafficked layer, the optimization of the texture is not relevant. Rather, the focus should be on improving porosity and flexibility. The elasticity is significantly influenced by the proportion of crumb rubber in the polyurethane asphalt layer. The more coarse rubber particles are added to the grain structure, the higher becomes the flexibility of the grain structure. This is because the flexibility of individual grains allows it to be compressed more under load. However, since rubber is the determining factor for the poor stability of the system, the rubber content must be reduced. Hence, this study proceeds in such a way that the proportion of rubber is reduced iteratively. For each test specimen produced in the first part of this study, the acoustic effectiveness is examined on the basis of the acoustic tube and the elastic modulus on the basis of an uniaxial pressure swelling tests.

**Figure 2:**
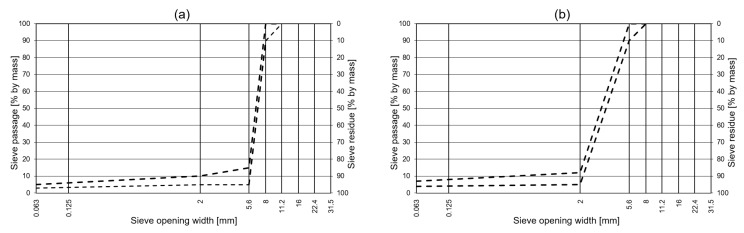
Grading curves according to the German guidelines of Porous Asphalt [20] (**a**) and [21] (**b**).

The porosity was tested in preliminary studies. These showed that test specimens with a void content of 35% by volume deliver good results. [19] confirmed this in his studies. Various grading curves were tested for the work. In order to use a statement for the selection of the maximum grain size of the grading curve, several possible grading curves with the maximum grain sizes of 5 mm and 8 mm were tested. In the end, it was found that the grading curves in [20,21] were suitable for the anticipated solution. The left grading curve [20] of Figure 2 is a grading curve from a german standard for an open-porous asphalt mix, which is conventionally used for road paving. The right grading curve [21] of Figure 2 is a grain composition for an open-graded mixture used on agricultural roads in Germany. It is usually used unbound and therefore offers a high permeability for the infiltration of rainwater. As these grading curves deliver open-graded mixtures with small grain sizes, it was decided to use them with partial replacement of the aggregates by rubber particles and a binder content of 6% by volume of the one component polyurethane.

The tested variants are shown in Table 1. As can be seen, the variant matrix is incomplete. A few specimens were reproduced in order to assess further results on elastic behavior with Var 8 specimens. It should be noted that no acoustic tests have been carried out on the Variants Var 8-2.5, Var 8-7.5 and Var 8-12.5. An exact composition of the polyurethane-bound test specimens is shown in Appendix A in Table A1.

#### 2.2.1. Impedance Measuring Tube

In this test method, the sound absorption coefficient of sound absorbers is determined using an impedance tube. The impedance measuring tube “AFD 1000—AcoustiTube” and the associated analysis software “AFD 1001—Determination of sound absorption coefficient according to DIN EN ISO 10534-2” [22] are used for this procedure.

**Figure 3:**
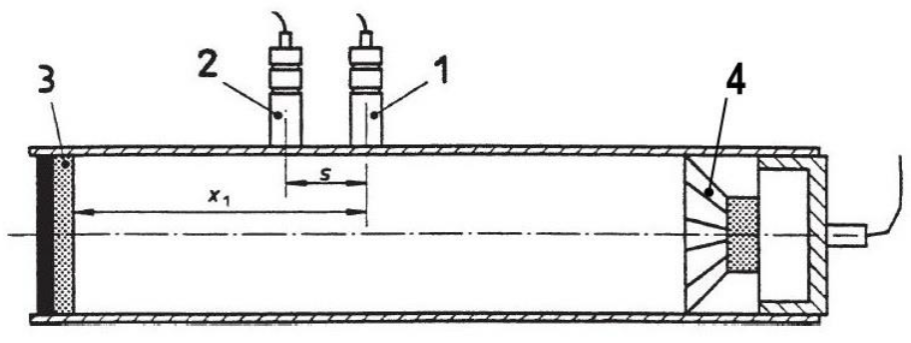
Impedance measuring tube according to [22].

The impedance tube (Figure 3) consists of a rigid and smooth cylindrical tube that is soundproof and airtight. At one end there is a loudspeaker (Figure 3, no. 4) and at the other end the specimen is positioned in a sample holder (no. 3). Two microphones are attached to the tube wall (no. 1 and 2). The diameter of the specimen is 100 mm, so that it fits exactly into the cross section of the tube. The specimen height of 40 mm is determined in such a way that it corresponds to the layer thickness in practice. By measuring the incident and reflected sound energy, the sound absorption coefficient is calculated and given as a function of frequency. The impedance tube used here covers the frequency range from 250 to 2000 Hz, whereby the respective sound absorption coefficient is determined in 3.125 Hz steps. Since this is a test method in which the specimens are not altered or damaged, each specimen is examined three times, the degrees of absorption of the measurements are averaged and this average value is given as the test result.

**Figure 4:**
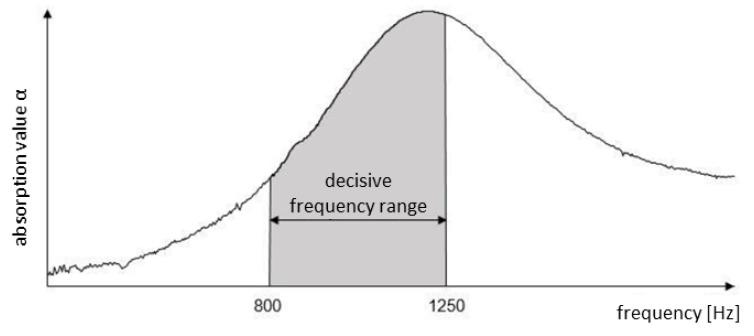
Decisive frequency range.

With regard to the target of reducing tyre–road noise, the sound pressure levels of the frequencies from 800 to 1250 Hz according to [4] are of particular importance. In the evaluation, the frequency-dependent absorption coefficients between these limits are considered (see Figure 4).

(1) $$\begin{array}{c} \alpha_{TRN}=\frac{\sum_{F_{u}}^{F_{o}}\cdot \alpha_{i}}{F_{o}-F_{u}}\\ absorption\;value:\alpha_{TRN}\\ lowest\;frequency=800\;Hz:F_{u}\\ highest\;frequency=1250\;Hz:F_{o}\\ absorption\;value\;at\;frequency\;i\;\left[-\right]:\alpha_{i} \end{array}$$

To convert the relevant sound absorption coefficients to a measurement or variant-specific value, they are averaged within this range, so that for each measurement one absorption value of the tyre–road noise can be given ($\alpha_{TRN}$).

#### 2.2.2. Uniaxial Loading Tests

The aim of this study is to create a flexible and durable absorption layer. As a binding agent, BASF’s 1-component polyurethane Elastan* 6568/103 is used, since it was developed for flexible floors, such as sports fields and playgrounds. It is a very elastic binding agent with excellent adhesion to rubber particles.

In order to evaluate the elasticity of the mixture as a whole, the modulus of elasticity can be evaluated. This is the elastic component of the material. The viscous part of the material behaviour is very low here and is therefore not used as a decisive factor for the evaluation.

For the experimental determination of the elastic modulus of asphalt, dynamic tests with uniaxial sinusoidal loading at a temperature of 20 ${}^{\circ }$C are used. The sinusoidal load enables the E-modulus to be divided into its elastic and viscous components as Equation (2) shows. This can be carried out by using swelling tests [23].

(2) $$\begin{array}{c} \left|E^{*}\right|=E^{\prime \prime }+E^{\prime }\\ storage\;modulus:E^{\prime }\\ loss\;modulus:E^{\prime \prime }\; \end{array}$$

Due to the elasticity of the specimens, the problem arose that the test stamp repeatedly lifted off the specimen during the test and the universal testing machine stopped frequently. By gradually increasing the contact stress from $\sigma_{u}=0.025$ to 0.06 N/mm${}^{2}$ it was possible to ensure that the stamp remains in contact with the test specimen. The upper contact stress was not changed ($\sigma_{o}=0.35$ N/mm${}^{2}$).

**Figure 5:**
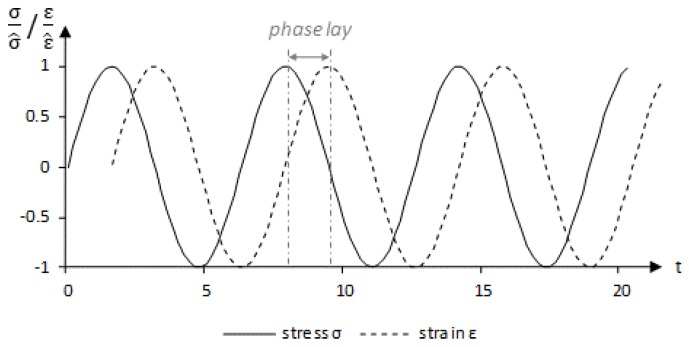
Sinusoidal loading according to [23].

The absolute Young’s modulus $\left|E^{*}\right|$ is determined by the continuous application of a dynamic sinusoidal voltage. Figure 5 shows a resulting strain reaction of a viscoelastic body. The strain is dependent on temperature, frequency and load.

The absolute Young’s modulus is calculated from the amplitudes of the stress $\hat{\sigma }$ and strain $\hat{\varepsilon }$ using Equation (3). In addition, the phase angle is calculated, which can be determined from the phase lay Δt between stress and strain amplitude or by the determination of the phase spectrum.

The result of the test is a creep curve with two phases, as Figure 5 is showing schematically. In detail, the force and displacement signals obtained from the uniaxial loading test were converted into stresses and strains and divided into their amplitude and phase spectra. This is possible through Fourier Transformation in Matlab^®^. The maximum amplitude of the stress $\hat{\sigma }$ and the strain $\hat{\varepsilon }$ and the corresponding phase angle ϕ can then be read off from the respective amplitude spectra and phase spectra at the load frequency. Hence, the absolute modulus $\left|E^{*}\right|$ is received from Equation (3).

(3) $$\left|E^{*}\right|=\frac{\hat{\sigma }}{\hat{\varepsilon }}$$

According to Equation (2) the elastic an viscous components of $\left|E^{*}\right|$ are derived from Equations (4) and (5).

(4) $$E^{\prime \prime }=E_{el}=\frac{\hat{\sigma }}{\hat{\varepsilon }}\cdot cos\phi$$

(5) $$E^{\prime }=E_{vis}=\frac{\hat{\sigma }}{\hat{\varepsilon }}\cdot sin\phi$$

#### 2.2.3. Testing of Fatigue Resistance

If a material is subjected to repeated loads, the resistance may be reduced by changing its internal stresses and its structure with each additional load. This results in a reduction in the strength of the material until failure occurs.

To determine the fatigue resistance, the method described in [24] is used throughout Germany. This method is intended for testing bituminous asphalt. Ref. [25] proposed a modified procedure for testing polyurethane-bound asphalt. The test is carried out analogously to the DIN standard, but the strain paths of the dynamic fatigue test are adapted as a function of the material. First, the static flexural strength of the material is determined on the basis of [24]. Then the dynamic test is conducted on the basis of the path-controlled three-point bending test based on [24] in order to determine the stiffness. The tests to determine fatigue resistance are performed at a uniform test temperature of 20 ${}^{\circ }$C.

To find out the static bending tensile strength, a prismatic specimen is integrated into the testing device. The load application device consists of two outer support rollers and an upper central roller for load transfer. The test specimen is mounted with its longitudinal axis perpendicular to the longitudinal axis of the rollers in the middle of the testing machine. After the specimen has been installed and tempered to 20 ${}^{\circ }$C within the testing machine, the test begins with a load controlled displacement by the upper support pressing on the specimen. The test is completed as soon as the test specimen fails. During deflection, the displacement and the applied force are measured to calculate the static flexural strength.

Following the static bending test, the dynamic three-point bending test according to [25] is performed to address the fatigue behavior. The method developed there is based on the procedure according to Annex C of [24]. In contrast to the original test, the strain amplitudes are determined as a function of the maximum deflection that was determined within the framework of the determination of the static flexural strength. The lower amplitude s_u_ remains the same as in the DIN standard. The upper amplitude, on the other hand, is set at 2/3 of the maximum deflection from the static flexural tensile test $s_{o}=\frac{2}{3}\cdot s_{max}$. This was found to be sufficient in the study conducted from [25]. The test setup, the specimens and the test temperature of the three-point bending test are identical to the conditions of the static bending test. The roller of the upper support is sinusoidally shifted between the vertices s_u_ and s_o_ at a frequency of 10 Hz and the force applied is measured. The test is terminated after 20,000 load cycles [24,25].

#### 2.2.4. Testing of Low Temperature Behavior

When asphalt is exposed to low temperatures, internal stresses—so-called cryogenic stresses—occur. In addition, the road material is usually subjected to traffic, which also creates tensions—the mechanogenic stresses. To determine these stresses, uniaxial tension stress tests (UTST) and thermal stress restrained specimen tests (TSRST) are carried out on the material. The UTST causes the formation of mechanogenic stresses, whereas the TSRST causes cryogenic stresses. The aim is to determine the tensile strength reserve, which forms the stress reserve from cryogenic and mechanogenic stresses. Which allows the determination of possible additional stresses that can be absorbed. Both tests require prismatic specimens which are connected to the testing device at their end faces.

During the UTST, the specimen is brought to a defined temperature within the test chamber. The specimen is then subjected to tensile stress and the resulting stresses and strains are continuously recorded. The test is terminated as soon as the specimen fails. The tensile force at which the specimen failed is used for the evaluation. This is converted into a stress via the cross-section of the specimen. The test is carried out at a total of four temperature levels (+20 ${}^{\circ }$C, +5 ${}^{\circ }$C, −10 ${}^{\circ }$C and −25 ${}^{\circ }$C) so that a tensile strength is determined for the common service temperature range.

The TSRST causes the formation of cryogenic tensions. While the specimen is clamped in the test device and kept at a constant distance, the temperature is continuously lowered (10 K/h). The cooling causes temperature-related stresses which are recorded by the test device as a function of the temperature. They are called thermally induced stresses [26].

## 3. Results and Discussion

### 3.1. Evaluation of the Suitable Variant

**Figure 6:**
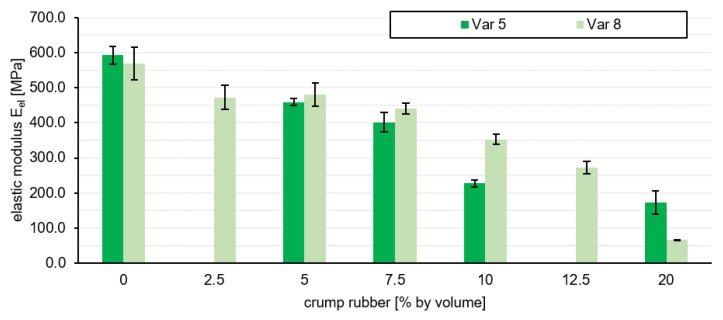
Evaluation of the elastic modulus of all tested variants.

The results of the tests mentioned above were evaluated in order to be able to make a decision for the preferred variant on the basis of this information. The pressure swelling test yielded the results shown in Figure 6. The evaluation of the layer performance is based on the elastic modulus of elasticity E_el_ in accordance to the best absorption properties. Three specimens of each variant have been tested. The final elastic modulus E_el_ used in the assessment is a mean value of the individual values.

The variants without crumb rubber have an elastic modulus $E_{el}$ of 592.2MPa (Var 5-0) and 568.8MPa (Var 8-0). The more aggregates are substituted by rubber granulate, the smaller is the E_el_ of both grading curve variants, which is 172.7MPa for Var 5-20 and 65.8MPa for Var 8-20. It can be stated that the addition of rubber granulate leads to a reduction in stiffness. The force is transmitted within the polyurethane asphalt layer via a supporting skeleton formed by the aggregates. Due to the volume-accurate, proportional substitution of the aggregates by crumb rubber, a part of the applied force is absorbed by the rubber granules.

**Figure 7:**
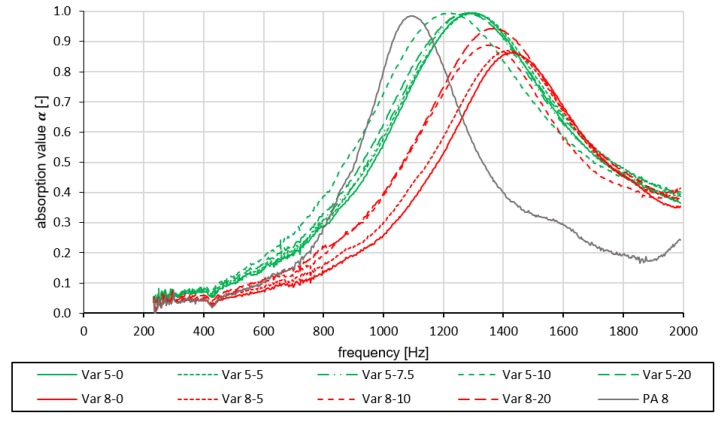
Absorption curves of all tested variants including a reference variant (PA 8).

Basically, it can be seen that the absorption curves of the variants tested in this study differ from those of a conventional porous asphalt PA 8. The PA 8-curve is integrated as a reference value in Figure 7 in order to compare it with the used mix variants from this study.

Looking at the test results in Figure 7, it can first be seen that the frequency bands of the sound absorption coefficient of the tested variants have pronounced maxima in the range from 1200 to 1300 Hz (Var 5) and from 1350 to 1450 Hz (Var 8). The maximum values of the absorption coefficient of Var 5 with α=0.99 to 1.00 are significantly higher than those of Var 8 (α=0.85 to 0.95). In addition, the absorption maximum of Var 5 covers a wider frequency range (α≥0.5 from approx. 900 to 1700 Hz) than the one of Var 8 (α≥0.5 from approx. 1100 to 1700 Hz). Both variants Var 5 and Var 8 have slipped higher in the frequency range compared to the reference variant and their curves cover a wider frequency range. The wider shape of the absorption curves indicates that the new material guarantees higher degrees of absorption in a larger frequency range.

When comparing the absorption coefficients of Var 5, no clear influence of the rubber content can be discerned due to the very similar curves. However, it is noticeable that the maximum of Var 5-10 is shifted by approx. 100 Hz to a lower frequency range compared to the other four curves. In comparison to Var 5, clear differences can be observed in the curves of Var 8 depending on the rubber content. By increasing the rubber content, increased maximum degrees of absorption can be achieved with α=0.925 (Var 8-10) and α=0.948 (Var 8-20). In addition, a dependency of the associated frequency on the rubber content is discernible, as it moves into a lower frequency range. An explanation is that the cavities of Var 5 have smaller diameters or locally smaller volumes due to the reduced proportion of coarse aggregates. The cavity system in the test specimens, which is responsible for the absorption of the incident sound, becomes smaller and closer meshed as a result. The enlarged inner surface of the polyurethane asphalt skeleton absorbs the incident sound more effectively. Watching the absorption curves of the different variants Var 5 and Var 8, it can be seen that the amplitudes of the absorption curves differ. This is a consequence of higher flow resistance which is characterized through Var 5 which forms a narrow and branched cavity system resulting from a smaller grain size distribution.

**Figure 8:**
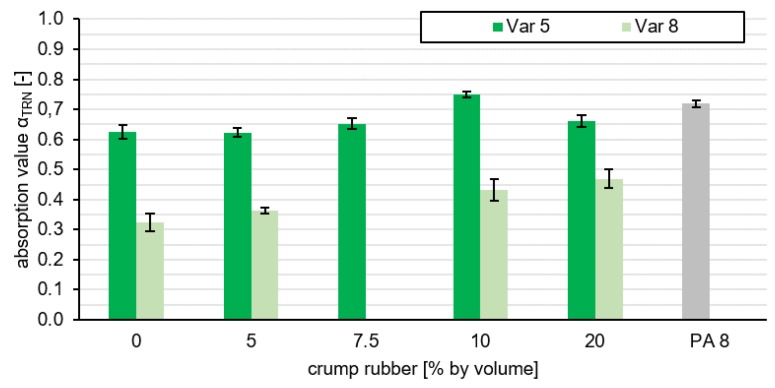
Representation of the absorption value via the proportion of crumb rubber.

In order to determine which variant is suitable both acoustically and in terms of durability, the proportion of space below the absorption curve in the range of 800–1250 Hz is used to evaluate all variants. The procedure was explained in Section 2.2.1. Finally, the normalization results in an absorption value for each tested variant $\alpha_{TRN}$. Figure 8 shows that Var 5 has higher absorption values compared to Var 8. Figure 8 describes the absorption value of the respective variant in dependence of the rubber content. From this it can be clearly deduced that the grain size has a decisive influence on the absorption potential of a road layer, because all absorption values of Var 5 are significantly higher than those of Var 8. The PA 8 was also examined with regard to its absorption value. In Figure 8, the layer PA 8 is regarded as the comparative value, which shows that the variant Var 5 examined here has very similarly good noise-reducing properties. Since Var 5 contains additional rubber particles, we expect a further noise-reducing effect due to the mechanical impedance effect. This, however, is not examined in this study.

**Figure 9:**
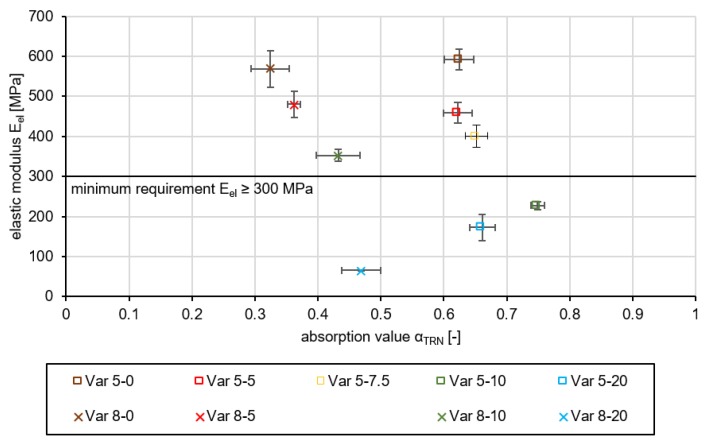
Representation of the elastic moduli across the absorption values of all tested variants.

In order to consider not only the acoustic effectiveness but also the durability, the calculated elastic moduli were plotted on the absorption values in Figure 9.

It can be seen that variants with a high absorption capacity have low elasticity values. There are also variants with a higher elastic modulus that have lower absorption values. Nevertheless, on the basis of Figure 9, a statement can be made about the variant that has the best absorption coefficient at the minimum requirement of E-modulus ≥ 300 MPa.

Due to higher and wider acoustic absorption of sound, it can be concluded that Var 5 has better acoustic properties than Var 8. One possible cause is the finer pored and nested structure of the cavity network caused by the smaller aggregates, which leads to a shift of the absorption maximum to a lower frequency and a higher flow resistance. However, Var 5-7.5 forms the highest acoustic efficiency with a stable design. For this reason, it is examined and evaluated by asphalt perfomance tests in order to see whether it is suitable for use in road traffic.

### 3.2. Asphalt Performance of Var 5

To test the performance of the developed material, test specimens with the composition of the final variant Var 5 were prepared. This is followed by tests on deformation behavior using the pressure swelling test, tests on fatigue resistance using the three-point bending test and tests on low-temperature behavior using the uniaxial tension stress test and the thermal stress restrained specimen tests. The specimens are produced in accordance with the dimensions required for testing and are shown in Table 2. In order to obtain the corresponding dimensions, plates were produced from which the test specimens were drilled or cut.

In order to evaluate the findings from this study, a comparison to values from the literature is conducted. The results of the variants tested by [25,28] were used for this purpose. In his studies, Ref. [25] compared the polyurethane-bonded mixtures PU-Var. A and PU-Var. B with regard to performance characteristics, in which only the size of the maximum grain size of the aggregates used is differentiated. PU-Var. A consists of a grading curve with a maximum grain size of 8 mm whereas PU-Var. B contains a maximum grain size of 5 mm.

#### 3.2.1. Pressure Swelling Test

The compression-swelling test according to TP Asphalt-StB [27] is carried out to test the deformation resistance of the material. The test specimen is loaded by a load stamp with a defined force over 10,000 cycles. The load is applied by a load pulse which applies a maximum stress of $\sigma_{o}=0.35$ MPa in a haversine-shaped course. During the loading pause the specimen is exposed to an undervoltage of $\sigma_{u}=0.025$. Since an unpublished preliminary study has shown that reacted polyurethane is nearly temperature-independent, the test temperature is reduced from 50 ${}^{\circ }$C to 20 ${}^{\circ }$C.

#### 3.2.2. Deformation Resistance

**Figure 10:**
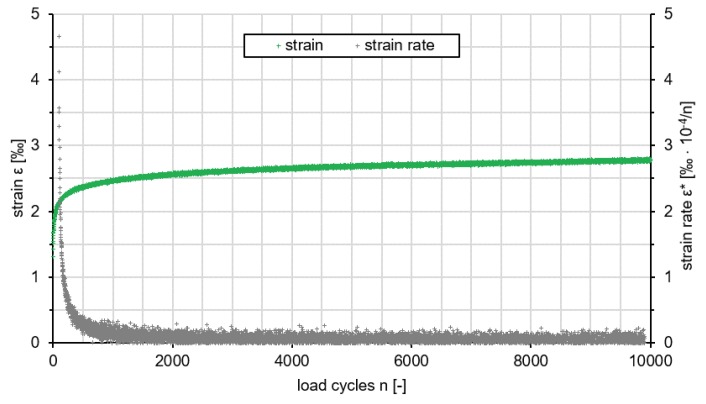
Results of the pressure swelling test and impulse creep curve (green) and strain rate (grey) of Var 5.

Strain and strain rate of Var 5-7.5 are shown in Figure 10 as a function of the load cycle. The strain of the variant has the typical course of a pulse creep curve without a turning point. There is no failure of the test specimens according to the recorded data. This was confirmed in the visual evaluation of the test specimen after loading, since no external damage was visible. After a stronger deformation at the beginning of the test, the strain rate decreases regressively ($\varepsilon^{*}=0.142$‰$\cdot 10^{-4/n}$ after load cycle 1000) and approaches asymptotically zero within the 10,000 load cycles ($\varepsilon^{*}=0.017$‰$\cdot 10^{-4/n}$ at the end of the test).

In comparison to reference variants PA 8 and PU-Var. B taken from [25], Var 5-7.5 has an extremely high resistance to deformation. While the PA 8 with a deformation of 90.42‰reaches the turning point at 263 load cycles and fails after about 750 load cycles, the strain rate of the Var 5-7.5 at the same time is only 2.28‰. Also, the associated strain rate (PA 8: approx. 2.2‰$\cdot 10^{-4/n}$) is clearly lower with 0.569‰ $\cdot 10^{-4/n}$. The tested PU-Var. B from [25] seems to have a similar course as the Var. 5-7.5 tested in this study. There is no turning point during the 10,000 load cycles and an deformation of approximately 10‰is achieved. It fails after about 40,000 load cycles.

The resistant material behavior of Var 5-7.5 is mainly based on the use of the polyurethane binder. This will possibly be supported by the highly regenerable rubber granulate. The complete coating of the aggregates with polyurethane results in a monolithic structure despite the open-graded structure of the polyurethane asphalt mix. The reduction of the strain rate over the increasing number of load cycles can be attributed to the fact that no binder-induced creep occurs with polyurethane-bound aggregates and that the test specimen can only be damaged due to higher loads. A modification (increased loads) has been made by [25] for this case to cause the material to fail. This has not been applied to the present material, yet.

In conclusion, it can be stated that the deformation properties of the investigated Var 5-7.5 proved to be very good. Compared to an porous asphalt PA 8, there is very little deformation.

#### 3.2.3. Results of the Three Point Bending Test

**Figure 11:**
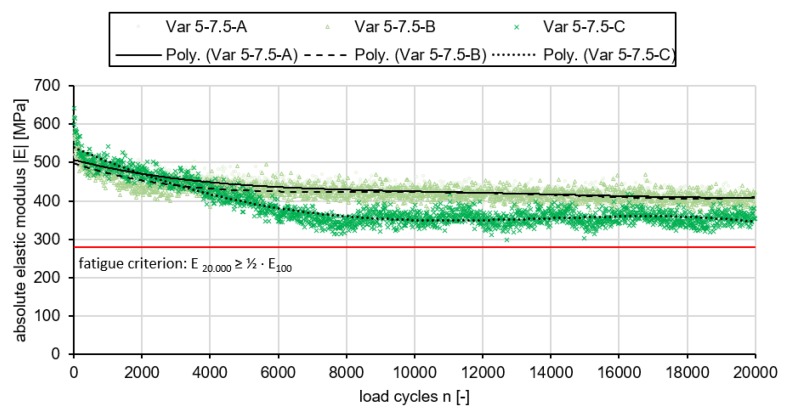
Results of the fatigue test; Stiffness decrease of three specimens of Var 5.

Following, the results of the three point bending test are shown in Figure 11. Three tests on the same polyurethane asphalt mix have been conducted. Looking at them, it is visible that two of the three (A and B) have an almost same course, while Var 5-7.5-C differs slightly. First, the absolute elastic modulus of the test specimens drops sharply to a relatively constant level and shows hardly any fatigue in the course of time. At Var 5-7.5-C, the elastic modulus sinks slightly longer until it also stabilizes at a certain level.

The fatigue test results are also shown in Table 3. A specimen is considered fatigued when the stiffness |E|, which decreases over the period of loading, drops to half the initial stiffness. The initial stiffness is the stiffness recorded at the 100th load cycle. The load cycle at which fatigue occurs is important for evaluation in order to draw a comparison of fatigue times between different mixes. For the evaluation a mean value of all tested variants (A, B and C) was taken as a basis. Compared to the initial stiffness after 100 load cycles, which at 537 MPa is significantly lower than that of the PU-Var. B (${\left|E\right|}_{100}=3322$ MPa) of [25], there is only a slight decrease in stiffness over the further load cycles. After 20,000 load cycles, the mean stiffness of all tested variants is 386.1 MPa and thus still corresponds to 72% of the initial stiffness ${\left|E\right|}_{100}$. No external damage or cracking could be observed on the test specimens after the end of the test.

The number of load cycles at which the value of the elastic modulus assumes half of its initial value cannot be determined, since the tests were terminated after the 20,000 load cycles, in accordance with the test procedure of the DIN standard.

In summary, it can be said that the newly developed material Var 5-7.5 has a fundamentally lower elastic modulus compared to other mix variants ([25,28]). However, the variant is characterised by its partial elasticity, which has decreased only slightly over the course of the tests carried out. Fatigue that occurred with other polyurethane-bound variants of [25] was not achieved by the variants tested in this study, although the test conditions adapted to the material were applied. Accordingly, the material has good fatigue resistance and is suitable for use in transport infrastructure.

#### 3.2.4. Results of the Uniaxial Tensions Stress Test and the Thermal Stress Restrained Specimen Test

To evaluate the low-temperature behavior of asphalts, a combined evaluation of UTST and TSRST is performed. The maximum tensile stresses $\beta_{t,max}$ are plotted on a cubic spline curve in a diagram as a function of temperature. The curve of thermal stresses $\sigma_{cry}$ of the material is integrated in the same diagram in order to determine the tensile strength reserve $\Delta \beta_{t}$ from the difference between the two curves.

**Figure 12:**
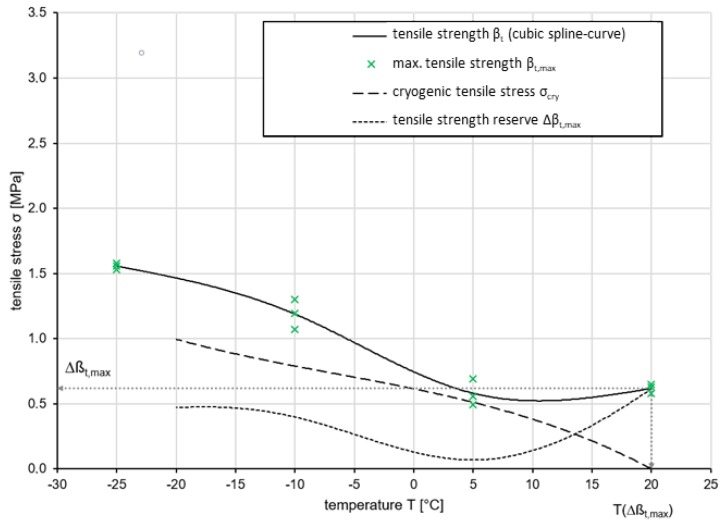
Results of the low temperature behavior of Var 5-7.5.

The cryogenic behavior of Var 5-7.5 is shown in Figure 12. It can be seen that the cryogenic tensile stress has its maximum of 0.62 MPa ($\sigma_{cry(T)}$) at 20 ${}^{\circ }$C, reaches a minimum at lower temperatures ($\sigma_{cry(T)}$(5 ${}^{\circ }$C) = 0.06 MPa) and rises again to $\sigma_{cry(T)}$(−17.7 ${}^{\circ }$C) = 0.48 MPa. The tensile strength reserve $\Delta \beta_{t}\left(T\right)$ resulting from the difference between the two input variables increases to 0.52 MPa at 5 ${}^{\circ }$C and doubles to −20 ${}^{\circ }$C (0.99 MPa).

It can be seen that the cryogenic tensile stress increases to 0.52 MPa at 5 ${}^{\circ }$C and doubles to −20 ${}^{\circ }$C (0.99 MPa). Its function $\sigma_{cry(T)}$ has an initially degressive and then linear course. The tensile strength reserve $\Delta \beta_{t}\left(T\right)$ resulting from the difference between the two input variables has its maximum of 0.62 MPa ($\Delta \beta_{t,max}$) at 20 ${}^{\circ }$C, reaches a minimum at lower temperatures ($\Delta \beta_{t}$(5 ${}^{\circ }$C) = 0.06 MPa) and rises again to $\Delta \beta_{t}$(−17.7 ${}^{\circ }$C) = 0.48 MPa.

On the basis of the measurement results, the low temperature behavior of Var 5-7.5 can be assessed as inadequate. Even at a temperature drop to 5 ${}^{\circ }$C, the tensile strength reserve for absorbing mechanogenic, i.e., traffic load-related tensile stresses drops to a critical level of 0.06 MPa. The tensile strength, which remains unchanged at this temperature, contrasts with increasing cryogenic stresses.

Therefore, the material has been modified in terms of its binder content. The polyurethane content would be increased from 6% by volume (2.2% by weight) to 13% per volume (5% by weight). This binder content was determined to be optimal in studies which tested several amounts of binder at low temperatures and it was determined in the study of [25] as well. An increase in the binder content considerably increases the tensile strength of the material, but prospectively does not limit its deformation and durability behavior, as the polyurethane-film merely wraps around the aggregate to form a monolithic system. Excess material concentrates at the bottom of the specimen.

Probably the monolithic structure at Var 5-7.5 was not completely formed, so that there were unglued areas in the aggregate structure. This circumstance could now be counteracted. Performance in terms of deformation and durability on the adjusted mixture should continue to be consistent if not better.

**Figure 13:**
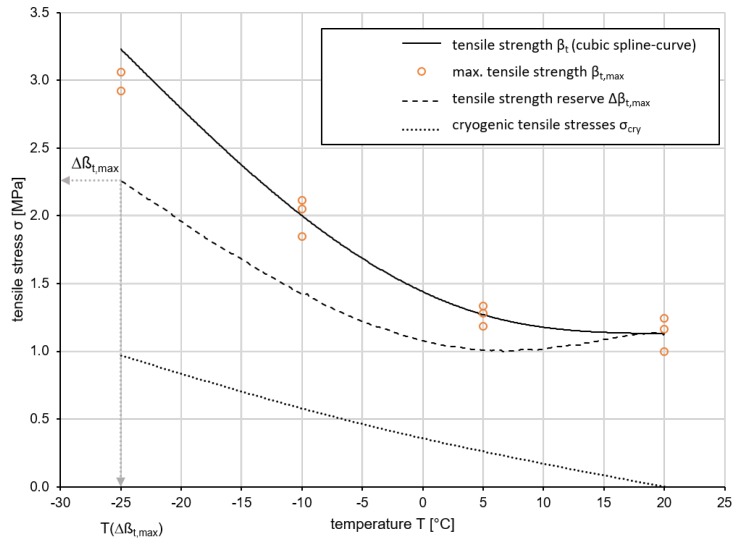
Results of the low temperature behavior of Var-5-7.5-T.

The low temperature behavior of Var 5-7.5-T is shown in Figure 13. In contrast to Var 5-7.5, Var 5-7.5-T has a significant higher tensile reserve, which is due to the high tensile strength of the material. By increasing the binding agent, the material can be compared with conventional asphalts and classified in an appropriate range.

A comparison of the variants from [25] with the characteristic values for low temperature behavior is made using the Table 4. The maximum tensile strength reserve and the corresponding temperature were used for the evaluation.

Var 5-7.5-T does not achieve higher tensile forces in the low temperature range than the polyurethane variant of [25] or the SMA 11 S [25]. However, the maximum tensile strength reserve values of the materials tested in this study occur at completely different temperatures. Especially cryogenic stresses decrease with increasing temperature. A difference to the PU-Var. A of [25] is the used crumb rubber and the elastified polyurethane in Var 5-7.5 and Var 5-7.5-T, which leads to a more elastified construction and thus, to lower tensile strength. A course that is common in conventional asphalt cannot be determined in the presented results. It should be noted that the low-temperature behavior of the comparative values was investigated by different testing institutes (University of Siegen and asphalt-labor, Arno J. Hinrichsen GmbH & Co.) and that therefore identical test conditions cannot be guaranteed.

## 4. Conclusions

In this study, an innovative noise-reducing pavement layer was developed that can be placed under a special surface layer. A material has been developed that reduces noise and is durable, which was problematic with comparable materials from previous studies ([1,3,4,7,14,15,17]). In order to achieve this goal, an iterative parameter study was carried out in which a potential material mixture was investigated by determining the elastic modulus and the absorption values. The best material composition was then subjected to tests to determine the performance characteristics in order to evaluate whether the polyurethane asphalt could be used for traffic infrastructure applications.

It could be proven that the developed material enables a 100% absorption of the incoming traffic noise in a defined frequency range. This absorption is made possible by a wide-branched cavity system in the layer. Compared to other tested variants, the optimal variant is characterized by a wider amplitude of the absorption curve. This means that the optimal variant can absorb more noise over a wider frequency spectrum than other variants. Hence, not only can noise of certain traffic collectives efficiently be absorbed, since it occurs in a defined frequency range, but the noise absorption can also work for changing traffic collectives. This is the result of a high flow resistance caused by narrow and rough pore spaces in the material, which are mainly due to the choice of the maximum grain size of 5 mm.

By testing the performance in terms of deformation resistance, fatigue resistance and low-temperature behavior, it can be shown that the material is suitable for use in road traffic. High deformation and fatigue resistances can be detected, which are given by a strong resilience of the tested specimens. The low-temperature behavior provides acceptable values.

In principle, it can be said that this material is qualified for use in road traffic with regard to noise reduction. The use of sustainable components and the integration of recycled rubber promotes the efficiency of building materials. This is because materials are recycled, which means conserving non-renewable resources. This is followed by a strengthening of the materials, which results in greater durability and saves the transport network many interventions due to repairs.

## Figures and Tables

**Table 1:** Tested variants.

Crumb Rubber	Max. Grain Size 5 mm	Max. Grain Size 8 mm
[% by Volume]	(Var 5)	(Var 8)
0	Var 5-0	Var 8-0
2.5	-	Var 8-2.5
5	Var 5-5	Var 8-5
7.5	Var 5-7.5	Var 8-7.5
10	Var 5-10	Var 8-10
12.5	-	Var 8-12.5
20	Var 5-20	Var 8-20

**Table 2:** Dimensions of tested specimens according to [24,26,27].

Performance Test	Shape	Diameter/Width × Depth	Height
pressure-swelling test	cylinder	100±5 mm	60±1 mm
three-point bending test	prism	40×40 mm${}^{2}$	320mm${}^{2}$
uniaxial tension stress test and
thermal stress restrained specimen test	prism	40×40mm${}^{2}$	160mm${}^{2}$

**Table 3:** Results of fatigue testing. PU-Var. A and B taken from [25].

Variant	${\left|E\right|}_{100}$	$n(1/2\cdot {\left|E\right|}_{100})$	${\left|E\right|}_{20,000}$
Var 5-7.5	537	not reached	386
PU-Var. A	2277	831	577
PU-Var. B	3322	4201	727

**Table 4:** Results of low temperature behavior of Var 5-7.5, Var 5-7.5-T from this study and PU-Var. A and SMA 11 S from [25].

Variant		Var 5-7.5	Var 5-7.5-T	PU-Var. A	SMA 11 S
T	[${}^{\circ }$C]	20	−25	−9.9	−6.5
$\Delta \beta_{t,max}$	[MPa]	0.618	2.261	3.248	3.397

## Abbreviations

The following abbreviations are used in this manuscript:

FGSV	Road and Transportation Research Association (Germany)
Var 5	Variant 5
Var 8	Variant 8
DIN	German institute for standardization (Deutsches Institut für Normung)
DIN EN	German institute for standardization (European standardization)
DIN ISO	German institute for standardization (International standardization)
PERS	Poro-elastic road surface
UTST	Uniaxial tension stress test
TRN	Tyre Road Noise
TSRST	Thermal stress restrained specimen test

## Appendix A

**Table A1:** Mixture composition of tested 5 mm variants in % by volume.

Aggregate	Fraction	Density	Var 5-0	Var 5-5	Var 5-7.5	Var 5-10	Var 5-20	Var 5-7.5-T
	[mm]	[g/cm^3^]						
limestone	filler	2.73	4	4	4	4	4	4
basalt	0–2	3.05	3	3	3	3	3	3
basalt	2–5	3.09	93	88	85.5	83	73	85.5
crumb rubber	super grob	1.1	0.0	5	7.5	10	20	7.5
polyurethane	Elastan	1.1	6	6	6	6	6	13
